# MOC31PE immunotoxin – targeting peritoneal metastasis from epithelial ovarian cancer

**DOI:** 10.18632/oncotarget.18694

**Published:** 2017-06-27

**Authors:** Yvonne Andersson, Synne Ihler Haavardtun, Ben Davidson, Anne Dørum, Karianne G. Fleten, Øystein Fodstad, Kjersti Flatmark

**Affiliations:** ^1^ Department of Tumor Biology, Norwegian Radium Hospital, Oslo University Hospital, 0424 Oslo, Norway; ^2^ Department of Pathology, Norwegian Radium Hospital, Oslo University Hospital, 0424 Oslo, Norway; ^3^ Department of Gynecologic Oncology, Norwegian Radium Hospital, Oslo University Hospital, 0424 Oslo, Norway; ^4^ Department of Gastroenterological Surgery, Norwegian Radium Hospital, Oslo University Hospital, 0424 Oslo, Norway; ^5^ Institute of Clinical Medicine, University of Oslo, 0310 Oslo, Norway

**Keywords:** MOC31PE immunotoxin, EpCAM, peritoneal metastasis of epithelial ovarian cancer, chemotherapy, animal models

## Abstract

Peritoneal metastasis (PM) is an important feature of epithelial ovarian cancer (EOC) and is a frequent site of drug resistant disease recurrence, identifying PM-EOC an important clinical challenge. The MOC31PE immunotoxin targets and kills tumor cells expressing the epithelial cell adhesion molecule (EpCAM), which is highly expressed in EOC, and MOC31PE is being investigated for use in treatment of PM-EOC.

The efficacy of MOC31PE treatment alone and in combination with cytotoxic drugs was investigated in two human EpCAM expressing EOC cell lines, B76 and MDHA-2774, *in vitro* and in corresponding mouse models mimicking PM-EOC. MOC31PE efficaciously killed tumor cells alone and showed equal or superior activity *in vitro* (paclitaxel, cisplatin, carboplatin) and *in vivo* (paclitaxel, mitomycin C) compared to the investigated cytotoxic drugs. Additive, or importantly, no antagonistic effects were observed in combination experiments.

In ex vivo cell culture, the cytotoxic effect of MOC31PE was studied on freshly isolated surgical EOC samples. All investigated fresh EOC samples expressed EpCAM and MOC31PE effectively reduced cell viability in ex vivo cultures.

In conclusion, these results, together with our previous preclinical and clinical experience, support development of MOC31PE for treatment of PM-EOC in combination with currently used cytotoxic drugs.

## INTRODUCTION

Epithelial ovarian cancer (EOC) is the leading cause of death from gynecological malignancies in the Western world. High-grade serous ovarian cancer (HGSOC) is the dominant histological subtype, comprising approximately 70% of cases. With poor anatomical barriers between the tissue of origin and the peritoneal cavity, peritoneal metastases (PM) are extremely common in EOC. Even in stage I disease, tumor cells may be present in peritoneal fluid samples, stage II comprises peritoneal tumors in the pelvic cavity and stage III includes PM to the remaining peritoneal surfaces, stages classification according to International Federation of Gynecology and Obstetrics (FIGO) [19]. In addition to being a common phenomenon in primary EOC, PM is an important feature of recurrent and end-stage disease. In general, patients initially respond well to standard treatment, which involves cytoreductive surgery (CRS) and systemic platinum/taxane combination chemotherapy. However, more than 75% of patients develop recurrent disease, and the 5-year survival is only 20-30% [14, 18]. An important feature of recurrent EOC is development of drug resistance; in particular, platinum resistance is strongly associated with poor prognosis [20, 21]. A challenge is therefore to develop improved therapies that will be efficacious in both the primary and metastatic settings, specifically targeting intraperitoneal tumor deposits with the aim of preventing and treating PM.

We have developed the MOC31PE immunotoxin in which the monoclonal antibody MOC31 is covalently linked to Pseudomonas exotoxin A (PE) [1, 2]. MOC31 binds the epithelial cell adhesion molecule (EpCAM), a transmembrane glycoprotein with high expression in carcinomas, including HGSOC, and with low expression in nonmalignant tissues [6, 16]. After binding to the surface of EpCAM-expressing tumor cells, MOC31PE is internalized and rapidly triggers cell death [1, 3] even in chemotherapy resistant cancer cells, broadening the potential utility of the drug. Recently, we demonstrated that systemic administration of MOC31PE was well tolerated in patients with EpCAM-expressing metastatic carcinomas [2]. In the human EOC cell lines B76 and HOC7 we demonstrated that MOC31PE decreased cell viability and cell migration *in vitro*, suggesting MOC31PE as a potential drug candidate in EOC [23].

In the present work, we have evaluated MOC31PE treatment alone and in combination with chemotherapeutic drugs in experimental models of human EOC. Two animal models mimicking the remaining minimal burden of disease in the peritoneal cavity after CRS were used to investigate the efficacy of intraperitoneal (i.p.) administration of MOC31PE and the cytotoxic effect of MOC31PE was analyzed in short-term cultures of surgical specimen obtained from patients with primary EOC, providing further support for use of MOC31PE in EOC.

## RESULTS

### *In vitro* efficacy of MOC31PE alone and in combination with chemotherapy

**Figure 1:**
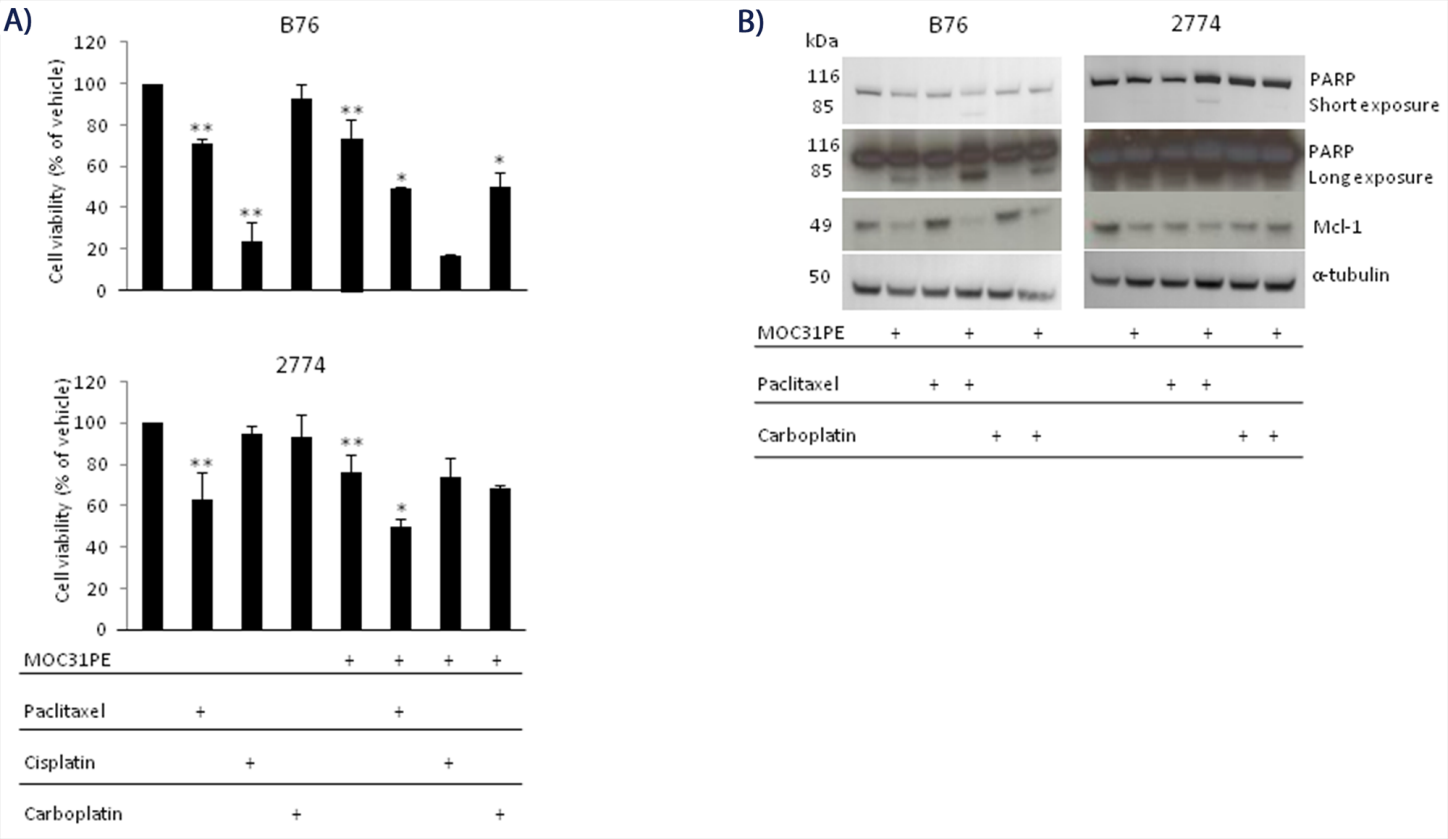
MOC31PE in combination with paclitaxel causes additive cytotoxicity *in vitro* **(A)** B76 and 2774 cells were incubated with paclitaxel (50 nM) and/or carboplatin (100 µM) and/or cisplatin (33 µM) and/or MOC31PE (10 ng/ml) for 24 h. Cell viability is expressed as a percentage (mean {plus minus} SD) of the value obtained in vehicle treated cells. The assays were performed in triplicate, and repeated at least three times. **; p<0.05 compared to vehicle treated cells, *; p<0.05 compared to monotherapies. **(B)** Western immunoblots of cell lysates from B76 and 2774 cells treated for 24 h with MOC31PE (10 ng/ml) and/or paclitaxel (50 nM) and/or carboplatin (100 µM). The 116- and 85-kDa bands represent the uncleaved and cleaved versions of PARP, respectively.

In the present experiments, the cytotoxicity of paclitaxel, carboplatin, and cisplatin, alone and in combination with MOC31PE was examined in B76 and 2774 cells (Figure 1A). The B76 cells were carboplatin resistant (IC50 was not reached even at 72h, data not shown), moderately sensitive towards paclitaxel (IC50 was not reached at 24h, Figure 1A) and IC50 was 25 nM at 72h (not shown) and cisplatin sensitive (IC50 16.5 μM, not shown). The 2774 cells exhibited moderate sensitivity to paclitaxel, but were resistant to both platinum compounds. In both cell lines, cell viability was reduced by 25-30% upon incubation with a low concentration of MOC31PE (10 ng/ml at 24h), and for combination experiments, this concentration of MOC31PE was used, which inhibited protein synthesis by 50% in both cell lines (Supplementary Figure 1 for 2774 and B76 [23]). The combination of MOC31PE and paclitaxel caused a 50% reduction in cell viability in both cell lines compared to the monotherapies (25-30%) (p<0.05), signifying an additive effect of combing the drugs. Co-treatment with MOC31PE and carboplatin reduced cell viability of B76 cells by approximately 20% compared to treatment with MOC31PE alone (p<0.05), but this effect was not observed in 2774. For the combination of MOC31PE and cisplatin, there was a nonsignificant trend towards reduced viability. Notably, the *in vitro* results show that MOC31PE cytotoxic effect was not antagonized in combination with standard chemotherapy.

Previously, we showed that induction of apoptosis is an important mechanism for the cytotoxic effect of MOC31PE [3] and in line with this MOC31PE resulted in partial inactivation of the DNA repair enzyme PARP (Figure 1B). Combining MOC31PE and paclitaxel further increased PARP inactivation (the lower band) in both cell lines. As expected, MOC31PE exposure reduced the levels of the anti-apoptotic protein Mcl-1 [3]. The single agents, paclitaxel and carboplatin had no effect on Mcl-1 in B76 but were effective in reducing Mcl-1 levels in 2774.

### Peritoneal metastasis models of human EOC

**Figure 2:**
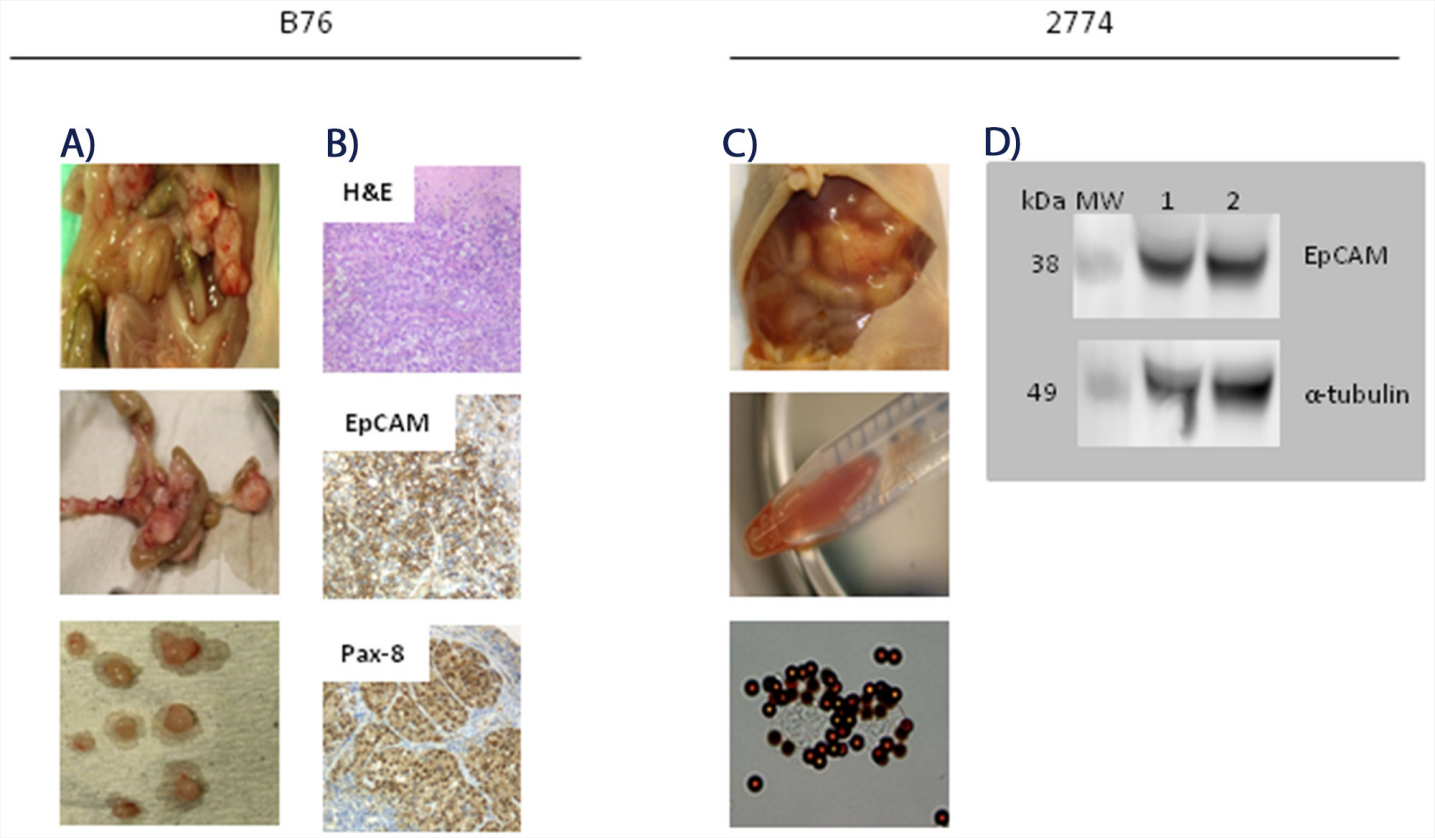
Characterization of peritoneal metastasis models of human epithelial ovarian carcinoma **(A)** Photographs taken at autopsy of a representative mouse on day 24 after i.p. injection of 2.5x10^6^ B76 cells. A mean number of 54 (SD 17) tumor nodules were seen distributed all over the peritoneal surfaces, including ovaries, kidneys, bladder, intestine and spleen. **(B)** Representative images of B76 tumor sections after hamatoxylin-eosin (H&E) and immunohistochemical staining. H&E stained B76 tumor section (top), strong EpCAM expression in the plasma membrane (middle), and both cytoplasmic and nuclear Pax8 staining (lower) picture. Magnification x40. **(C)** Photographs taken at autopsy of a mouse on day 23 after i.p. injection of 2.5x10^6^ 2774 cells. A mean number of 25 (SD 12) tumor nodules with a distribution similar to that seen with B76 cells. EpCAM-positive cancer cells in the ascites fluid were detected and isolated with immunomagnetic MOC31-coated beads (two lower pictures). **(D)** Immunoblots showing EpCAM expression (lane 1 and 2) in isolated 2774 cells from ascites of two representative animals with anti-α-tubulin antibody as loading control.

The B76 cell line was established from a patient with serous, chemotherapy-naïve HGSOC, and we present the first results with this cell line in an animal model. The i.p. B76 model proved to be very robust with a 100% take rate, and the mean survival time (MST) was 24 days (SD 1.0). The main signs of i.p. tumor growth were abdominal distension without ascites and weight loss. At autopsy on day 24, a mean number of 54 (SD 17) tumor nodules were observed distributed on the peritoneal surfaces; on the parietal peritoneum, major omentum and mesentery, on the ovaries, kidneys, bladder, intestine and spleen (Figure 2A). Tumor tissues were analyzed with respect to expression of a range of proteins with relevance for characterizing EOC subtypes and to discriminate between EOC and malignant mesothelioma [9, 16]. The tumor tissues were positive for expression of EpCAM, PAX8, B72.3, CLD3, MUC4, and CK7, whereas calretinin, WT-1, CK20, CDX2 and Villin were not detected. The positive IHC results for membrane bound EpCAM and intense nuclear PAX8, and H&E-staining are shown in Figure 2B (not all data shown). The tumor marker profile and morphological characteristics are suggestive of HGSOC and compatible with the original patient’s tumor tissue [17].

The 2774 cell line was established from a patient with endometrioid EOC, and this model was also robust with a 100% take rate, and mice developed ascites with distended abdomen (Figure 2C), leading to sacrifice after a mean of 23 days (SD 1.3). The mean number of tumor nodules detected was 25 (SD 12) and the mean volume of ascites was 3.9 ml (SD 2.3). The mouse ascites contained EpCAM-positive cancer cells as demonstrated by IMS, also confirmed by strong EpCAM staining on western blots (Figure 2D). Histological examination and IHC marker profile were consistent with the cell line origin (data not shown).

### *In vivo* efficacy of MOC31PE

**Figure 3:**
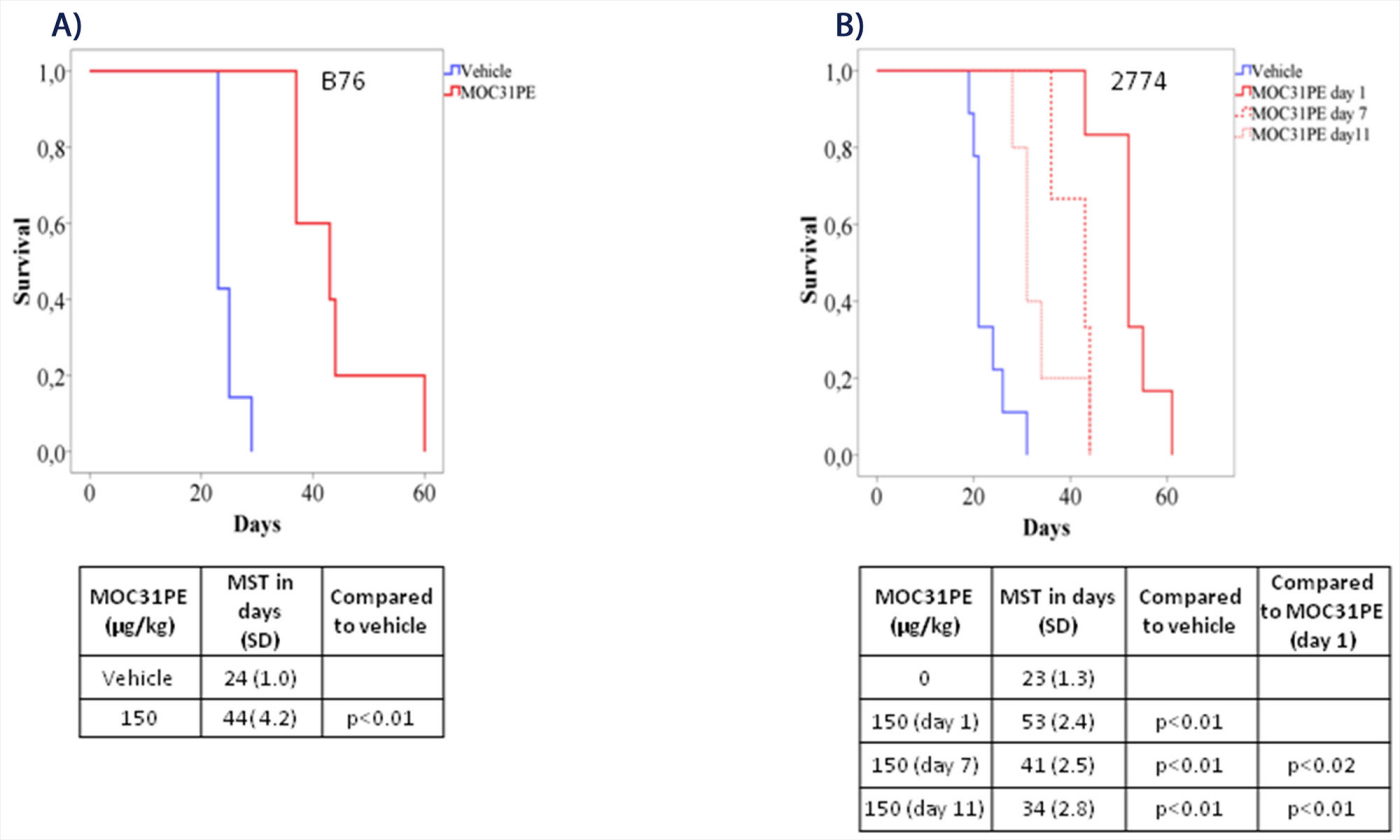
MOC31PE increases survival of mice with B76 and 2774 peritoneal metastases Kaplan-Meier survival curves of mice injected i.p. with **(A)** B76 and **(B)** 2774 cells. B76 animals were treated i.p. 24 h later with one dose of MOC31PE (150 µg/kg) or vehicle whereas 2774 mice received a single treatment dose either day 1, day 7 or day 11 after injection of the tumor cells. The mean survival time (MST) is summarized in the tables under the graphs.

Administration of MO31PE (150 μg/kg) i.p. significantly prolonged survival in the B76 model compared to vehicle treated animals, with a MST of 44 days (SD 4.2) versus 24 days (SD 1.0), respectively (p<0.01; Figure 3A). This dose of MOC31PE was well tolerated with no signs of toxicity. In the 2774 model, MOC31PE i.p. (150 μg/kg) increased survival significantly (53 days, SD 2.4) compared to vehicle (23 days, SD 1.3, (p<0.01)) (Figure 3B). In addition, a reduced ascites accumulation were record, with only 2 out of 6 animals presenting with measurable amount of ascites (3.7 ml and 2 ml), whereas all 6 vehicle treated mice had ascites (3.9 ml, SD 2.3). Notably, administration of MOC31PE (150 μg/kg i.p.) as a single treatment dose, significantly prolonged the MST of the treated mice was compared to the vehicle group, (p<0.01) whether the treatment was started on day 7 (41 days, SD 2.5) or on day 11 (34 days, SD 2.8) (Figure 3B).

### *In vivo* efficacy of MOC31PE in combination with chemotherapy

**Figure 4:**
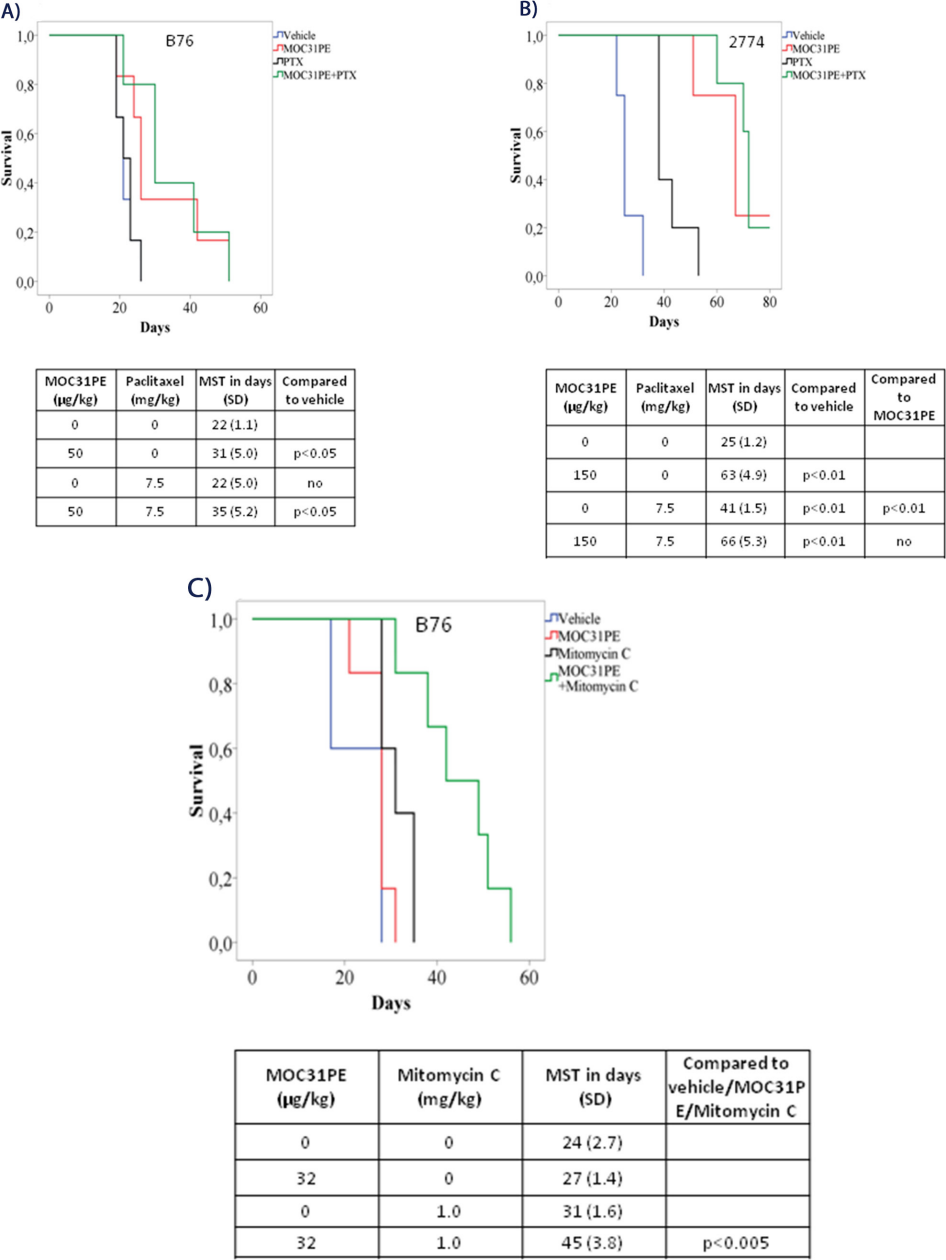
*In vivo* efficacy of MOC31PE, paclitaxel and mitomycin C as single agents and in combination experiments Kaplan-Meier survival curves of groups of at least five mice injected with B76 **(A)** or 2774 **(B)** cells and treated i.p. 24 h later with single doses of MOC31PE and paclitaxel (7.5 mg/kg) alone and in combination, with vehicle as control. The MOC31PE dose was low in B76 mice (50 µg/kg) and high in 2774 mice (150 µg/kg). The mean survival times (MST) and p-values are summarized in the tables under the graphs. **(C)** Kaplan-Meier survival curves of groups of at least five mice inoculated with B76 cells and treated i.p. 24 h later with single doses of a low MOC31PE (32 µg/kg) and a low mitomycin C (1mg/kg) or the combination, and with vehicle as a control. The MST summary for the different groups is shown in the table under the graph. The significance between treatment and vehicle animals were determined by the log-rank test.

Paclitaxel (7.5 mg/kg) treatment alone did not influence animal survival (22 days, SD 5.0) in the B76 model compared to vehicle (22 days, SD 1.1, p<0.05), while a low dose of MOC31PE (50 μg/kg) was still efficacious (31 days, SD 5.0) (Figure 4A). Combining MOC31PE and paclitaxel significantly increased MST (35 days, SD 5.2) with an efficacy similar to MOC31PE alone.

In the 2774 mice, paclitaxel (7.5 mg/kg) alone increased animal survival (41 days, SD 1.5) compared to vehicle (25 days, SD 1.2, p<0.01). The combination of MOC31PE (150 μg/kg) and paclitaxel was significantly more efficacious than vehicle (66 days, SD 5.3, p<0.01) and paclitaxel alone, but did not improve survival compared to MOC31PE alone (150 μg/kg) (63 days, SD 4.9) (Figure 4B). The experiment was terminated at day 80.

Based on our previous findings in animal models with PM-CRC [11], a low dose of MOC31PE (32 μg/kg) in combination with mitomycin C (1 mg/kg) was chosen also for the PM-EOC B76 model (Figure 4C). The MST of the combination treated mice (45 days, SD 3.8) was significantly (p<0.005) prolonged compared to that obtained with any of the single agents; MOC31PE 32 μg/kg (31 days, SD 1.6) and mitomycin C 1 mg/kg (27 days, SD 1.4), and vehicle (24 days, SD 2.7).

### Ex vivo efficacy of MOC31PE

**Figure 5:**
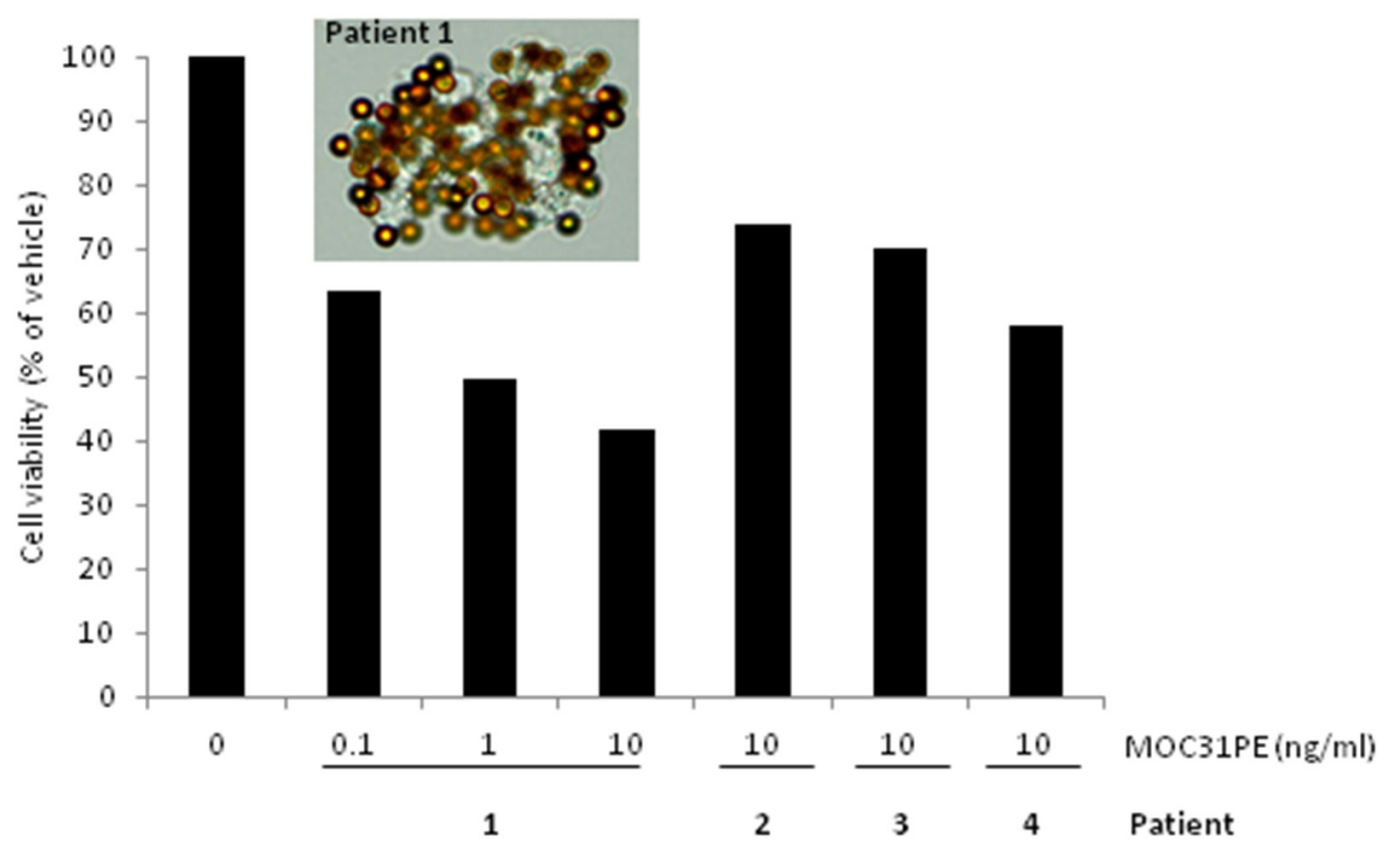
Ex vivo effects of MOC31PE on ovarian cancer cells freshly isolated from patients’ peritoneal metastases The picture shows EpCAM-positive cells isolated from disaggregated tumor tissue from patient 1 using immunomagnetic MOC31-coated beads. Cells from patient 1 were treated with MOC31PE (0.1-10 ng/ml) or vehicle and incubated at 37°C for 24 h, demonstrating a dose-response relationship with less than 40% cell viability at 10 ng/ml. Disaggregated tumor tissue from patient number 2, 3 and 4 was treated with MOC31PE (10 ng/ml), with cell viability of 60 and 75% compared to vehicle (p<0.05). The assays were performed in at least six parallels

To investigate MOC31PE cytotoxicity in more heterogeneous EOC cell populations than the cell lines represent, peritoneal tumor samples from four patients were taken directly from the operating theatre and prepared for short-term ex vivo culturing. All samples exhibited EpCAM expression as assessed by IMS using MOC31-coated immunomagnetic beads (Figure 5), Cells from patient 1 were treated with MOC31PE (0.1-10 ng/ml) or vehicle for 24h, demonstrating a dose-dependent reduction of cell viability with less than 40% cell viability at 10ng/ml (Figure 5). Generally, the tumor cell yield was low, limiting the possibility for analysis of a range of doses, time points and drugs. MOC31PE (10 ng/ml; 24h) resulted in reduced cell viability in all examined samples compared to vehicle treated cells, with 74%, 70%, and 68% remained cell viability for samples 2, 3, and 4, respectively (Figure 5). The 24 h incubation time point was chosen because a longer exposure period would have killed all EpCAM-expressing cells and potential differences in response between patients would not be possible to detect.

## DISCUSSION

In ovarian carcinoma, CRS is usually conducted with the aim of achieving complete removal of all macroscopically visible tumor tissue. Our models of PM-EOC attempt to mimic the peritoneal cavity after CRS, in which minimal residual disease, single tumor cells or small clusters of cells, represent the targets for therapeutic interventions. Therefore, in most experiments, animals were given i.p. injections of the drugs the day after injection of EOC cells. In well-tolerated doses, a single injection of MOC31PE improved survival and inhibited tumor growth in both models, while paclitaxel was shown to be efficacious in 2774 and had no significant effect in B76. No synergistic or additive effects were detected when paclitaxel was combined with MOC31PE *in vivo*, which is in contrast to the results from the *in vitro* experiments where at least additive effects were demonstrated. The results underline the importance of evaluating *in vitro* findings in more clinically relevant model systems. Importantly, the antitumor effects of MOC31PE were maintained in combination with paclitaxel in both *in vivo* models, the combination was well tolerated in the doses used, and no antagonistic effects were observed.

Combining MOC31PE and mitomycin C in doses that did not influence survival as single agents significantly increased survival in the B76 animal model, demonstrating at least an additive effect. This is in line with our previous results in experimental animal models of PM-CRC, showing that combining single i.p. injections of MOC31PE and mitomycin C inhibited tumor growth and prolonged survival [11], and mitomycin C could therefore be an interesting drug to consider for i.p. treatment in PM-EOC. Although systemic administration of mitomycin C has been almost completely abandoned because of toxicity, it emerges as an interesting drug in this context based on its use as a well-tolerated component of HIPEC in the treatment of PM-CRC [13]. The clinical use of mitomycin C in the treatment of EOC is not well explored. When administered i.p. or as component of HIPEC alone, or in combination with other drugs, mitomycin C has exhibited low toxicity and disease control has been reported in some cases [5, 7, 8, 15]. Mitomycin C could represent an alternative to cisplatin as component of HIPEC in EOC, reducing the risk of renal impairment, which is a concern with cisplatin. How to most efficaciously combine MOC31PE and mitomycin C as components of HIPEC is currently being examined in our PM-EOC animal model systems.

In this study, EpCAMs presence on the surface of cells from HGSOC tumors was confirmed by IMS in freshly prepared cell suspensions, and treatment with MOC31PE caused reduction in cell viability. Interestingly, one of the four cases in this study had a *BRCA-1* mutation, which is associated with hereditary EOC of the HGSOC subtype, and the effects of MOC31PE exposure were similar in this case compared to the wild-type cases. For unknown reasons, EpCAM has been shown to be especially highly expressed in metastatic and recurrent, chemotherapy resistant EOC [6]. Therefore, attention has been on developing drugs targeting EpCAM, particularly for use in a palliative setting, but the few candidates that are currently available are unfortunately associated with severe side effects. Our clinical experience with MOC31PE suggests our drug to be very well tolerated when administered systemically [2]. In addition, MOC31PE has a competitive advantage by a “simpler” mode of action compared to other anti-EpCAM antibody based therapeutics, requiring only binding to EpCAM-expressing cancer cells, after which it directly induces death of cancer cells, through release of the toxin inside the target cells.

In conclusion, treatment with MOC31PE increased survival of mice in models of PM-EOC, and at least additive effects were observed on survival and tumor growth in combination with mitomycin C, which is a drug commonly used as a component of HIPEC in PM-CRC. Furthermore, a low dose of MOC31PE effectively reduced cell viability of tumor cells extracted directly from freshly harvested PM-EOC samples. These results together with our previous experience with MOC31PE in preclinical and clinical studies encourage further evaluation of MOC31PE in the treatment of PM-EOC.

## MATERIALS AND METHODS

### Cells

The human EOC cell lines B76 [17] and MDHA-2774 (2774) [12, 17] were a gift from Dr C. Marth (Innsbruck Medical University, Innsbruck, Austria). Cells were grown at 37°C in RPMI-1640 medium supplemented with Hepes, Glutamax (all from Lonza, Austria), 8 % heat-inactivated FCS (PAA, GE Healthcare, UK), and Estradiol, named complete RPMI-1640 medium. All cell lines were routinely tested and found to be free from contamination with Mycoplasma species. The cells and xenograft tissues were routinely ID tested.

### Drugs

MOC31PE immunotoxin was prepared as previously described [4]. Paclitaxel, cisplatin and carboplatin were from Sigma Aldrich (St. Louis, MO) and mitomycin C was from Medac (Wedel, Germany). For the *in vitro* assays, the drugs were diluted in complete RPMI 1640 medium, and for *in vivo* experiments in 0.9% saline (B.Braun, Melsungen, Germany).

### Cell viability

The Cell Titer 96 AqueousOne solution assay (MTS (Promega Madison, WI)) was used to determine cell viability as previously described [4]. Cells (15 000 for B76 and 10 000 for 2774) were seeded in 96-well plates. After 24h incubation, the medium was replaced with medium containing MOC31PE (0.1-100 ng/ml), cisplatin (10-110 μM), carboplatin (5-500 μM), paclitaxel (2.5-1000 nM) or a combination of MOC31PE with paclitaxel/carboplatin/cisplatin and incubated further for 24h or 72h. The 72h treatment period was added to ensure at least two cell-doubling times. The assays were performed in triplicate, and repeated at least three times for the cell lines.

### Western immunoblotting

Cells (2x10^6^) were seeded in T-25 flasks and after 24h, treated with drug or vehicle for 24h, and handled as previously described [1]. Ascites collected from animals bearing 2774 xenografts was centrifuged and cells collected and protein lysates prepared [19]. Proteins were separated on 4-12% NuPAGE Bis-Tris gel (Invitrogen, Carlsbad, CA) and subsequently transferred by electrophoresis to Immobilon membrane (Millipore, Bedford, MA). The blots were probed with antibodies according to the manufacturers’ protocols. The antibodies used were anti-α-tubulin and anti-Poly (ADP-ribose) polymerase (PARP) (Calbiochem, La Jolla, CA) and anti-Mcl-1 (Santa Cruz Biotechnology, Santa Cruz, CA). Bound antibody was detected with HRP-conjugated secondary antibodies (Dako) and Super Signal West Dura substrate (Pierce, Thermo Scientific, Waltham, MA) in a G-Box/CCD camera system from SynGene.

### *In vivo* studies

All procedures and experiments involving animals were approved by The National Animal Research Authority and carried out according to the European Convention for the Protection of Vertebrates used for Scientific Purposes. Female mice (Athymic Nude-Foxn1^nu^), age 4-6 weeks, were kept under specific pathogen-free conditions, and food and water were supplied ad libitum, supplemented with 17-β-estradiol (4 mg/l). Tumor cells (2.5x10^6^) in medium without FCS were injected i.p. on day 0. Mice were randomly assigned to treatment groups (commonly 6 animals/group), and the chemotherapeutic drugs (paclitaxel and mitomycin C) or vehicle were administered in the form of a single injection i.p. (0.5 ml) on day 1, day 7, or day 11. In both models in the peritoneal cavity, no visible tumor nodules were detected at day 1; at day 7, most tumor nodules were 1-2 mm; and at day 11, larger tumor nodules of variable size were present. Day 1 was the starting point for treatment for most of the experiments, which was intended to reflect the condition in patients after CRS with only microscopic tumor lesions present in the peritoneal cavity. MOC31PE was administered i.p. 6 h after the chemotherapeutic drugs. The maximum tolerated dose (MTD) of MOC31PE given i.p. in mice was previously shown to be 150 μg/kg [11]. The paclitaxel dose used i.p. in combination with MOC31PE was 7.5 mg/kg. The well-being of the mice was carefully monitored, and animals were sacrificed by cervical dislocation if and when signs of disease and/or weight loss ≥ 15% were detected, or at the end of the experiment (defined to be at least 3x the observation period of untreated animals). At autopsy, metastatic lesions and ascites were collected, counted and weighed. Tumor tissues were formalin fixed, paraffin-embedded, sectioned, and haematoxylin-eosin (H&E) stained, and the presence of typical tumor tissue was verified.

### Immunohistochemistry

Formalin-fixed, paraffin-embedded sections from the peritoneal xenografts were examined as previously described [10]. Visualization was achieved using the EnVision + peroxidase system (Dako, Glostrup, Denmark). The antibodies used were against EpCAM and MUC4 (Abcam, Cambridge, UK), as well as Ber-EP4, carcinoembryonic antigen (CEA), calretinin, WT-1, CK20, CK7 and CDX2 (Dako). In addition the antibodies PAX8 (Abnova, Taipei City, Taiwan), B72.3 (BioGenex, Fremont, CA), Claudin-3 (CLD3) (Zymed, San Fransisco, CA), and Villin (Immunotec, Indianapolis, IN) were used. Negative control consisted of sections that underwent similar staining procedures with nonrelevant rabbit immunoglobulins or a monoclonal antibody of the same isotype as the primary antibody used. For all antibodies, positive controls were included with satisfactory results. The study pathologist (BD) evaluated immunohistochemical staining.

### Immunomagnetic selection

Ascites from tumor-bearing animals was analyzed for the presence of EpCAM positive cells by immunomagnetic selection (IMS) as previously described [22]. Briefly, immunomagnetic beads (Dynabeads M450 rat anti-mouse IgG1) coated with MOC31 anti-EpCAM antibody (IQ Products, Groningen, the Netherlands), or uncoated beads for control experiments, were added to 2x10^5^ live cells isolated from ascitic fluid. A cell was classified as EpCAM positive when at least five beads/cells were bound to the cell surface. No rosetted cells were observed in control experiments with uncoated beads.

### Short-term cultures of human peritoneal tumor samples

The use of human samples was approved by the regional ethics committee of South-East Norway and written informed consent was obtained from the patients (REK 2014/473). Peritoneal tumor samples were harvested at the time of surgery from patients with high-grade serous EOC, FIGO stage IIIc before receiving first-line chemotherapy, six cycles of paclitaxel and carboplatin, postoperatively. Three patients had wild-type BRCA-1. Patient number 3 had a *BRCA-1* mutation. Tumor samples were disaggregated and cells (10 000) were seeded in at least six parallels into 96-well plates for short-term cultures (24h) to assess efficacy of MOC31PE or vehicle as described above. In addition, the cells were analysed by IMS for the presence of EpCAM expression.

### Statistical analyses

Student’s t-tests were performed to compare treatment groups in the *in vitro* assays. Survival was estimated by the Kaplan-Meier method and groups were compared using the log-rank test. P-values <0.05 were considered significant. All analyses were performed using SPSS (Statistical Package for Social Sciences) version 21.0 (IBM, Chicago, IL, USA).

## SUPPLEMENTARY MATERIALS FIGURE


