# Supplementary material for: MOC31PE immunotoxin – targeting peritoneal metastasis from epithelial ovarian cancer
Source: Oncotarget. 2017 Jun 27;8(37):61800–9. doi: 10.18632/oncotarget.18694 (PMC5617465; doi:10.18632/oncotarget.18694)
Supplement: Supplementary file 1 [file oncotarget-08-61800-s001.pdf]

MOC31PE immunotoxin – targeting peritoneal metastasis from epithelial ovarian cancer

SUPPLEMENTARY MATERIALS

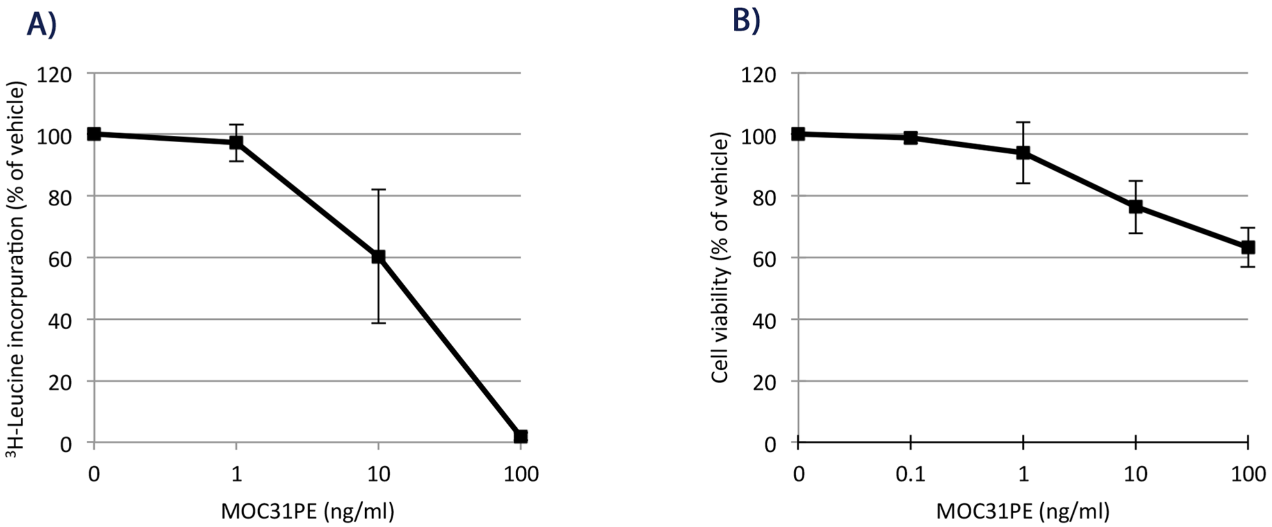

Supplementary Figure 1: The inhibitory effect of MOC31PE immunotoxin on protein synthesis and cell viability of 2774 cells. **(A)** Tumor cells were seeded in 48-well plates and the next day the medium was changed and MOC31PE added. Protein synthesis was analyzed after 24 h, by measuring the amount of [3H]-leucine protein incorporation. Values for treated cells are shown as the percentage of the values obtained in vehicle treated cells. The experiment was repeated twice. **(B)** For the cell viability test, cells were seeded in 96-well plates and MOC31PE added as described above. Mean {plus minus} SD of three independent experiments is shown with at least triplicate wells.
